# Effect of recombinant LH supplementation timing on clinical pregnancy outcome in long-acting GnRHa downregulated cycles

**DOI:** 10.1186/s12884-022-04963-x

**Published:** 2022-08-09

**Authors:** Chenyang Huang, Xiaoyue Shen, Jie Mei, Yanxin Sun, Haixiang Sun, Jun Xing

**Affiliations:** 1 Center for Reproductive Medicine and Obstetrics and Gynecology, Nanjing Drum Tower Hospital, Nanjing University Medical School, Nanjing, 210008 China; 2 Center for Molecular Reproductive Medicine, Nanjing University, Nanjing, 210008 China; 3 Drum Tower Clinic Medical College, Nanjing Medical University, Nanjing, 210008 China

**Keywords:** rLH supplementation timing, Long-acting GnRHa, Clinical pregnancy rate, Early miscarriage rate

## Abstract

**Background:**

Timely and moderate luteinizing hormone (LH) supplementation plays positive roles in in vitro fertilization/intracytoplasmic sperm injection and embryo transfer (IVF/ICSI-ET) cycles with long-acting gonadotropin-releasing hormone agonist (GnRHa) pituitary downregulation. However, the appropriate timing of LH supplementation remains unclear.

**Methods:**

We carried out a retrospective cohort study of 2226 cycles at our reproductive medicine centre from 2018 to 2020. We mainly conducted smooth curve fitting to analyse the relationship between the dominant follicle diameter when recombinant LH (rLH) was added and the clinical pregnancy outcomes (clinical pregnancy rate or early miscarriage rate). In addition, total cycles were divided into groups according to different LH levels after GnRHa and dominant follicle diameters for further analysis.

**Results:**

Smooth curve fitting showed that with the increase in the dominant follicle diameter when rLH was added, the clinical pregnancy rate gradually increased, and the early  miscarriage rate gradually decreased.

**Conclusions:**

In long-acting GnRHa downregulated IVF/ICSI-ET cycles, the appropriate timing of rLH supplementation has a beneficial impact on the clinical pregnancy outcome. Delaying rLH addition is conducive to the clinical pregnancy rate and reduces the risk of early miscarriage.

**Supplementary Information:**

The online version contains supplementary material available at 10.1186/s12884-022-04963-x.

## Background

Luteinizing hormone (LH) is a glycoprotein gonadotropin (Gn) produced by the adenohypophysis. Female LH mainly cooperates with follicle stimulating hormone (FSH) to promote follicular development, and plays an important role in inducing oocyte maturation, ovulation and luteinization. In addition, LH can promote androgen production, to maintain the synthesis and secretion of oestrogen and progesterone [1]. Therefore, FSH and LH supplementation seems to be essential for follicular development, embryo implantation, and the persistence of pregnancy [2–4]. Given the important role of LH in follicular development and hormone synthesis, whether exogenous LH should be added to controlled ovarian hyperstimulation (COH) cycle has been widely discussed [5–7].

At present, the long-acting gonadotropin releasing hormone agonist (GnRHa) for pituitary downregulation is widely used for in vitro fertilization/intracytoplasmic sperm injection-embryo transfer (IVF/ICSI-ET) [8, 9]. Two weeks after the injection of long-acting GnRHa in normal women of childbearing age, endogenous hormones are almost completely inhibited, and the FSH level gradually recovers from the 3rd-4th week, while the inhibition of the level of LH may last until the 8th week [10]. The long-term inhibition of LH can prevent the early LH peak in IVF/ICSI-ET cycles. However, the deep suppression of serum LH levels caused by long-acting GnRHa may adversely affect follicular development, hormone synthesis and oocyte quality, and impair the final clinical pregnancy outcome [11, 12]. Some studies have suggested that LH supplementation has a beneficial effect on the clinical pregnancy rate [13–15]. In contrast, other studies did not find added value of LH addition in IVF/ICSI-ET cycles [15–20]. Thus, it is impossible to draw a definite conclusion. The main reason for the inconsistent results is that there are too many uncertain confounding factors, such as the difference in patients’ ovarian response, the type of added LH, and the timing and dose of LH supplementation [7, 21–23].

This study explored the effect of recombinant LH (rLH) on the clinical pregnancy outcome at different periods of long-acting GnRHa downregulated IVF/ICSI-ET cycles to provide a theoretical basis for further clinical randomized controlled trials, which could provide a practical scheme for the addition of rLH in long-acting GnRHa IVF-ET cycles.

## Methods

### Basic information of the study population

This study was a retrospective analysis of patients who received long-acting GnRHa in vitro fertilization/intracytoplasmic sperm injection-embryo transfer (IVF/ICSI-ET) cycles with rLH addition at the reproductive medicine centre of Nanjing Drum Tower Hospital from January 2018 to December 2020. Each couple was aware that their data during assisted reproductive technology (ART) treatment might be used in this study. The study was approved by the ethics committee of Nanjing Drum Tower Hospital. The exclusion criteria were as follows: (1) ICSI cycles with sperm collected by testicular sperm aspiration (TESA) or percutaneous epididymal sperm aspiration (PESA); (2) cycles initiated with special type of FSH; (3) cycles of other kinds of exogenous LH addition; (4) cancelled cycles or cycles in which oocytes were frozen; and (5) cycles in which embryo transfer was cancelled for various reasons (ovarian hyperstimulation syndrome (OHSS), endometrial abnormalities, hydrosalpinx, personal factors, etc.). A total of 2226 cycles were included in our study.

### Controlled ovarian stimulation program

Long-acting GnRHa (decapeptide, triptorelin acetate, 1.875 mg or 3.75 mg, Ferring GmbH, Germany) was administered in the early follicular phase. After 28–42 days, the levels of serum oestrogen (E_2_), FSH, LH and progesterone (P) were measured. In addition, the diameter and number of follicles were monitored by transvaginal ultrasound. The criteria for pituitary downregulation were FSH < 5 mIU/mL, LH < 5 mIU/mL, and E_2_ < 30 pg/mL, and most follicles were 4.5–5 mm in diameter. Subsequently, 75–300 IU recombinant FSH (rFSH, Gonal-F, Merck Sereno, Switzerland) was injected every day, and 75–150 IU rLH (Luveris, Merck Sereno, Switzerland) was added as appropriate. The initial dosage of Gn was determined by the patient's age, body mass index (BMI) and anti-Mullerian hormone (AMH) level. The drug dose was adjusted according to follicular size and serum hormone levels (FSH, LH, E_2_ and P). Human chorionic gonadotropin (hCG, 10,000 IU) (Chorionic Gonadotrophin for Injection, Livzon Pharm, China) was injected to trigger oocyte maturation when the diameter of 1–2 dominant follicles reached 18 mm. For patients with a high risk of OHSS, 250 μg recombinant hCG (rhCG, Merck Sereno, Switzerland) or 5000 IU hCG was used. Oocytes were collected 36–38 h after triggering. Two pronuclei (2PN) appearing after fertilization of mature oocytes (metaphase II, MII) was considered normal fertilization. The quality of cleavage-stage embryos was evaluated from three aspects: cell number, fragmentation and symmetry. High-quality cleavage-stage embryos had 8–10 cells, a fragment proportion of less than 5%, and symmetrical blastomeres. Blastocysts were rated by using the Gardner scoring system. High-quality blastocysts had more than III stages of cystic expansion, and the rating of the inner cell mass (ICM) and trophectoderm (TE) was at least grade B [24]. The available embryo rate refers to the ratio of the number of high-quality cleavage-stage embryos to the number of total embryos. Some cleavage-stage embryos were cultured to the blastocyst stage.

### Embryo transfer and pregnancy detection

All patients included in this study received abdominal ultrasound-guided embryo transfer on the 3rd or 5th day after oocyte retrieval. After pregnancy, routine luteal support continued until two months after embryo transfer. Patients with a positive serum β-hCG level (more than 200 mIU/mL) measured 12–14 days after embryo transfer were scheduled to receive vaginal ultrasound examination of the pregnancy sac 30 days after embryo transfer. Patients with a lower serum β-hCG level (less than 200 mIU/mL) received a retest of the serum β-hCG level. Clinical pregnancy was defined as the presence of a gestational sac. The major outcomes measured in our study were clinical pregnancy and early miscarriage rate. The clinical pregnancy rate was the ratio of the number of clinical pregnancy cycles to the total number of embryo transfer cycles and the early miscarriage rate was the proportion of early miscarriage cycles to total clinical pregnancy cycles.

### Statistical analysis

The relationships between the dominant follicle diameter when rLH was added and the clinical pregnancy rate or the proportion of available embryos were analysed by smooth curve fitting. We used the Kolmogorov–Smirnov normality test to detect the normal distribution of variables. A t test was used for normally distributed variables, and the Mann–Whitney U test was used for nonnormally distributed variables. For the statistical analysis of categorical variables, the chi-square test was adopted (meeting the requirements of chi-square test: theoretical frequency (T) > 5 and sample number (n) > 40). The parameters with a normal distribution are presented as the mean ± standard deviation (SD). All analyses were performed with R (http://www.R-project.org) and EmpowerStats software (www.empowerstats.com, X&Y solutions, Inc. Boston MA). *P* < 0.05 was considered statistically significant.

## Results

**Fig. 1:**
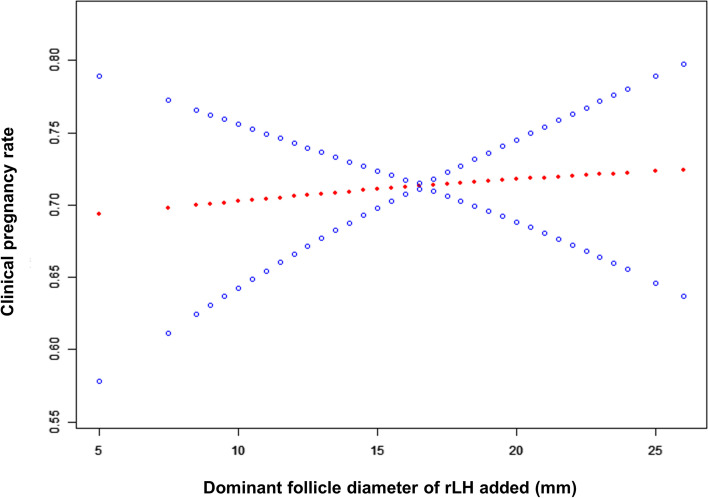
**A** smooth fitting curve analysis between dominant follicle diameter when rLH added and clinical pregnancy rates. The illustrated curved line shows the relation between the dominant follicle diameter when rLH added and clinical pregnancy rates. The area between two dotted lines is expressed as the 95% CI. The clinical pregnancy rate of the patients increased gradually as the dominant follicle diameter when rLH added increased

**Fig. 2:**
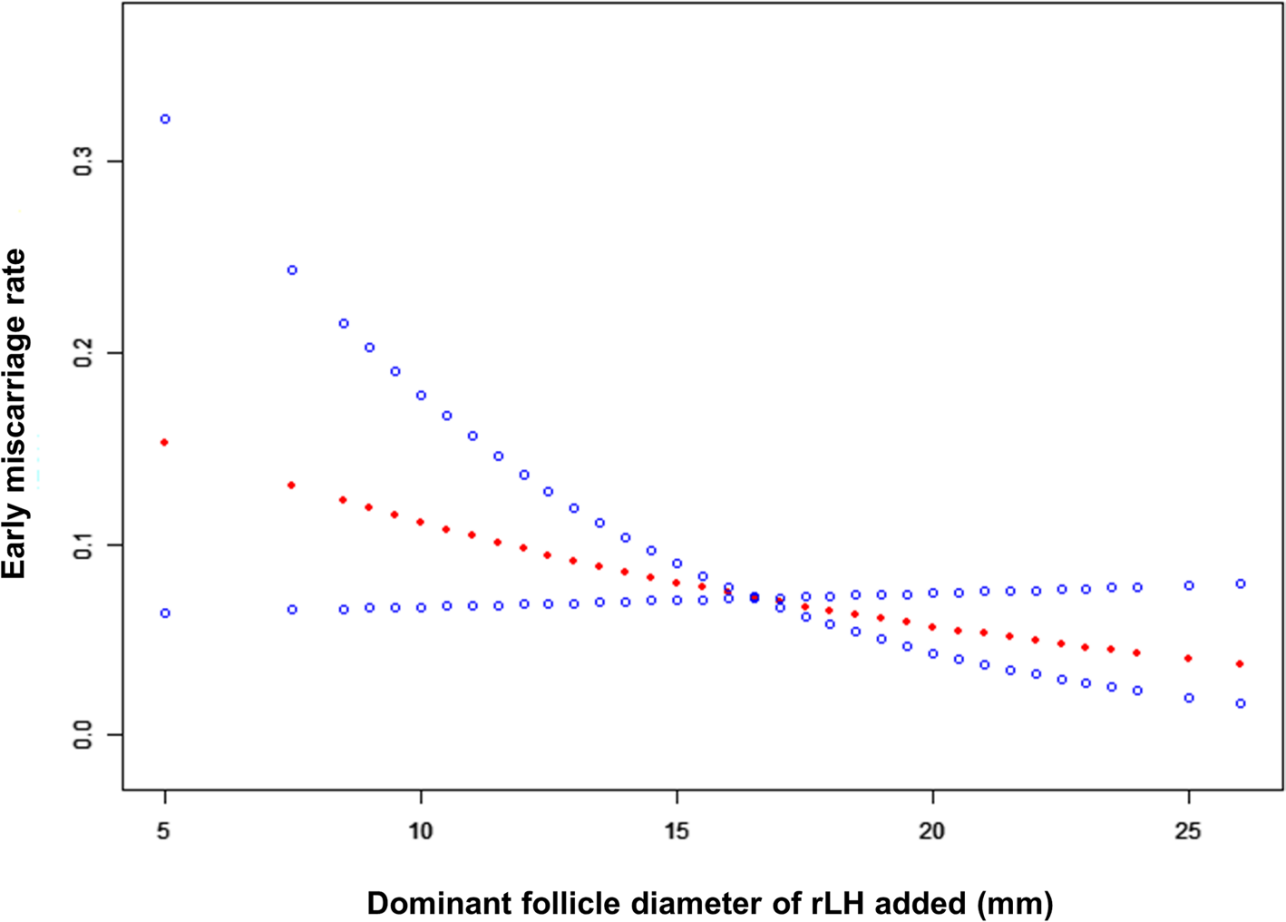
A smooth fitting curve analysis between dominant follicle diameter when rLH added and early miscarriage rates. The illustrated curved line shows the relation between the dominant follicle diameter when rLH added and early miscarriage rates. The area between two dotted lines is expressed as the 95% CI. The early miscarriage rate of the patients decreased obiviously as the dominant follicle diameter when rLH added increased

As shown in Table 1, the general data of all the enrolled patients and the relevant data regarding the IVF/ICSI-ET cycles were first statistically analysed. To explore the relationship between the timing of rLH supplementation and the final clinical pregnancy outcome, we conducted smooth curve fitting analysis. As shown in Fig. 1, the clinical pregnancy rate gradually increased with increasing dominant follicle diameter when rLH was added. In addition, we analysed the early miscarriage rate. The smooth curve fitting results suggested that with a delay in the timing of rLH supplementation (larger dominant follicle diameter at the time of adding rLH), the miscarriage rate decreased gradually (Fig. 2). Therefore, late rLH supplementation has a beneficial impact on the clinical pregnancy outcome of patients receiving long-acting GnRHa IVF/ICSI-ET cycles.

**Table 1:** General data of all enrolled patients and relevant data in IVF/ICSI-ET cycles

Female age (y)	29.79 ± 3.72
BMI (kg/m^2^)	23.13 ± 3.24
Infertility duration (y)	3.27 ± 2.15
Infertility type	
Primary infertility	54.99% (1224/2226)
Secondary infertility	45.01% (1002/2226)
Infertility factors	
Tubal factor	67.21% (1496/2226)
Ovulatory obstacle	17.25% (384/2226)
Reproductive tract	0.09% (2/2226)
Endometriosis or adenomyosis	2.20% (49/2226)
Male factors	8.22% (183/2226)
Unexplained infertility	4.81% (107/2226)
Basal FSH (mIU/mL)	7.09 ± 1.76
Basal LH (mIU/mL)	6.17 ± 4.04
Basal E_2_ (pg/mL)	44.17 ± 51.57
AFC (n)	20.54 ± 5.24
Initiated Gn dose (IU)	129.27 ± 34.76
FSH after GnRHa (mIU/mL)	3.46 ± 1.44
LH after GnRHa (mIU/mL)	0.64 ± 0.38
Total Gn dose (IU)	1966.18 ± 613.15
Gn duration (y)	12.04 ± 2.39
E_2_ on hCG day (pg/mL)	2892.95 ± 1324.46
LH on hCG day (mIU/mL)	1.61 ± 0.78
P on hCG day (ng/mL)	0.64 ± 0.40
Em (mm)	12.00 ± 2.54
Total rLH dose (IU)	192.66 ± 102.63
No. of of retrieved oocytes (n)	12.12 ± 3.69
No. of MII oocytes (n)	10.60 ± 3.57
MII rate	0.88 ± 0.13
No. of fertilized oocytes (n)	9.99 ± 3.54
No. of normally fertilized oocytes (2PN) (n)	9.06 ± 3.37
2PN rate	0.91 ± 0.11
No. of available embryos (n)	5.17 ± 2.47
Available embryo rate	0.59 ± 0.21
No. of transferred embryos (n)	1.56 ± 0.50
Type of transferred embryo	
cleavage-stage embryo	81.45% (1813/2226)
blastocyst	18.55% (413/2226)
No. of implanted embryos (n)	1.29 ± 0.48
OHSS rate	8.221% (183/2226)
Clinical pregnancy rate	71.29% (1587/2226)
cleavage-stage embryo transfer	70.49% (1278/1813)
blastocyst transfer	74.82% (309/413)
Early miscarriage rate	7.18% (114/1587)
cleavage-stage embryo transfer	7.04% (90/1278)
blastocyst transfer	7.77% (24/209)
Live birth rate	63.16% (1406/2226)
cleavage-stage embryo transfer	62.49% (1133/1813)
blastocyst transfer	66.10% (273/413)

## Discussion

LH is essential for follicular development and maturation. Some scholars previously proposed the “LH window” theory [25], suggesting that LH levels that are too high or too low have adverse effects on follicular development. Levels of LH that are too high will inhibit granulosa cell division, prematurely start follicular meiosis, stop follicular development, and lead to early atresia or luteinization of follicles. On the other hand, low LH levels will lead to insufficient oestrogen synthesis and a low response of granulosa cells to FSH, which will affect the maturation of the oocyte-corona-cumulus complex and luteal growth. In addition, low LH results in poor endometrial hyperplasia, increased dosage of Gn, decreased E_2_ levels on the hCG day, and decreased number of oocytes and fertilization rate. The conversion of pregnenolone to androgen and E_2_ is limited, which affects the concentration of E_2_ in follicular fluid. Pregnenolone accumulates in the body and impairs endometrial receptivity, which reduces the pregnancy rate and increases the risk of early miscarriage [26, 27].

GnRHa was used for pituitary downregulation in 1984 [28], and it has been increasingly widely used. However, due to individual differences such as age and ovarian reserve, patients may have different ovarian responses to standardized administration [29], and the degree of pituitary downregulation is difficult to accurately assess. GnRHa can inhibit more than 90% of LH levels and 40%-60% of FSH levels, which leads to excessive pituitary inhibition. The low serum LH level after downregulation affects follicular development, maturation and clinical outcomes [30]. At present, many studies [31–35] have shown that appropriate exogenous LH supplementation has a positive effect. However, there is no consensus on the suitable population and appropriate time to add exogenous LH. Most previous studies employed 0.5 mIU/mL as the criterion for the LH level for the overinhibition of the pituitary [36, 37]. We grouped the data according to this cut-off value. The results suggested that when the serum LH level after GnRHa downregulation was less than 0.5 mIU/mL, the addition of rLH had no additional benefit. There was no significant difference in the proportion of available embryos or clinical pregnancy rate between the two groups (Table. S1). Therefore, this study focused on the relationship between the timing of rLH addition and clinical outcomes.

This study found that the clinical pregnancy rate of patients who received long-acting GnRHa downregulated IVF/ICSI-ET cycles increased gradually with the increase in the dominant follicle diameter when rLH was added. The results suggest that delaying the addition time of rLH is beneficial to the clinical pregnancy outcome. Each follicle has an upper limit LH level. The expression of LHR on the surface of granulosa cells of nondominant follicles was low, and the premature LH increase easily exceeded the upper limit. Therefore, LH has a negative selective effect on nondominant follicles. The threshold of dominant follicles with healthy growth increased significantly. Especially after follicle selection, the expression of LHR in preovulatory follicles has been shown to be 10 times higher than that in antral follicles with diameters of 3–10 mm [38, 39]. When the diameter of dominant follicles is more than 14 mm, FSH induces a sudden increase in LHR on granulosa cells and intimal cells of dominant follicles under the synergistic effect of oestrogen. In the late follicular phase, LH partially replaces FSH, which is conducive to the selection and maintenance of dominant follicles. Therefore, LH addition in the late follicular phase is in accordance with the physiological state. We further analysed the timing of rLH supplementation, and divided all the data into two groups with a follicle diameter of 14 mm as the cut-off value. The results showed that when rLH was added after the diameter of the dominant follicle exceeded 14 mm, the clinical pregnancy rate and live birth rate were higher and the miscarriage rate was lower than those in the early addition group (Table. S2), but the differences were not statistically significant.

We found that the number of retrieved oocytes and the number of mature oocytes in the rLH late-addition group increased significantly, suggesting that late addition could increase the number of retrieved oocytes, but there was no significant difference in the proportion of MII oocytes. Some studies suggested that LH addition can promote the secretion of many growth factors in follicular fluid, which might interact with steroids, to protect oocytes from degeneration and allow appropriate nuclear maturation. Although LH supplementation might not increase the total number of oocytes obtained, it could improve the quality of retrieved oocytes and embryos [40]. Therefore, we further analysed the proportion of available embryos, and there was no significant difference between these two groups. The smooth curve fitting results showed that when the diameter of the dominant follicle was small, the proportion of available embryos was higher (Fig. S1). On the one hand, the number of cycles with early addition of rLH in this study was low (only 0.7% of dominant follicles had a diameter of ≤ 10 mm), so there might be individual differences. On the other hand, studies have shown that high or low LH supplementation will lead to poor endometrial hyperplasia and affect the pregnancy outcome [41]. Early addition of rLH might not benefit the endometrium but might lead to its abnormal function and impair embryo implantation. Therefore, the clinical pregnancy rate of patients who received early addition of rLH decreases. In addition to the exploration of the clinical pregnancy rate, some reports have suggested that the miscarriage rate of patients with higher serum LH levels after GnRHa downregulation is significantly higher [37, 42]. Similarly, our study found that after the premature addition of rLH, the early miscarriage rate was higher, and with the delay of rLH supplementation, the miscarriage rate decreased gradually.

However, there are some deficiencies in this study. Many studies have suggested that rLH supplementation in elderly women improves the embryo implantation rate and clinical pregnancy rate [43–45]. In addition, the addition of rLH helps prevent the early increase in progesterone levels in elderly patients [14, 46–49]. Moreover, some studies have suggested that rLH supplementation improves the number of oocytes obtained and the clinical pregnancy outcome in patients with a low ovarian response [14, 50, 51]. Most patients who received long-acting GnRHa downregulation at our centre were less than 35 years old and had normal ovarian reserve. Therefore, our study did not explore the timing of rLH addition in elderly and low ovarian response patients, which would be a further direction to be explored. In addition, studies have shown that the addition of both rLH and HMG has a beneficial effect on the pregnancy rate [15]. Our study only included rLH supplemented cycles, but many patients received HMG supplementation. Although HMG contains LH activity, it comes from hCG rather than LH. Therefore, we did not simultaneously evaluate the effect of HMG addition in our analysis. The most important defect is that this was a retrospective study. Randomized clinical trials with more samples are needed to further clarify the appropriate timing of rLH addition.

## Conclusion

In conclusion, our study found that in long-acting GnRHa downregulation cycles, when rLH was added when the dominant follicle diameter was larger, the clinical pregnancy rate increased and the early miscarriage rate decreased. Therefore, delaying the timing of rLH addition may benefit patients receiving these cycles.

## Supplementary Information

**Additional file 1:** **Figure S1.** A smooth fitting curve analysis betweendominant follicle diameter when rLH added and availableembryo rates. The illustrated curvedline shows the relation between the dominant follicle diameter when rLH addedand available embryo rates. The area between two dotted lines is expressed asthe 95% CI.

**Additional file 2:** **Table S1.** Comparison of different LH afterGnRHa.

**Additional file 3:** **Table S2.** Comparison ofdifferent dominant follicle diameter of rLH added groups.

## Data Availability

The datasets used and/or analysed during the current study are available from the corresponding author on reasonable request.

## Abbreviations

LH: Luteinizing hormone
IVF/ICSI-ET: in vitro Fertilization/intracytoplasmic sperm injection and embryo transfer
GnRHa: Gonadotropin-releasing hormone agonist
rLH: Recombinant LH
Gn: Glycoprotein gonadotropin
FSH: Follicle stimulating hormone
COH: Controlled ovarian hyperstimulation
ART: Assisted reproductive technology
TESA: Testicular sperm aspiration
PESA: Percutaneous epididymal sperm aspiration
OHSS: Ovarian hyperstimulation syndrome
E_2_: Oestrogen
P: Progesterone
BMI: Body mass index
AMH: Anti-Mullerian hormone
hCG: Human chorionic gonadotropin
2PN: Two pronucleus
MII: Metaphase II
SD: Standard deviation
