# Supplementary material for: Effect of recombinant LH supplementation timing on clinical pregnancy outcome in long-acting GnRHa downregulated cycles
Source: BMC Pregnancy Childbirth. 2022 Aug 9;22:632. doi: 10.1186/s12884-022-04963-x (PMC9364622; doi:10.1186/s12884-022-04963-x)
Supplement: Supplementary file 1 — Additional file 1: Figure S1. A smooth fitting curve analysis betweendominant follicle diameter when rLH added and availableembryo rates. The illustrated curvedline shows the relation between the dominant follicle diameter when rLH addedand available embryo rates. The area between two dotted lines is expressed asthe 95% CI. [file 12884_2022_4963_MOESM1_ESM.docx]

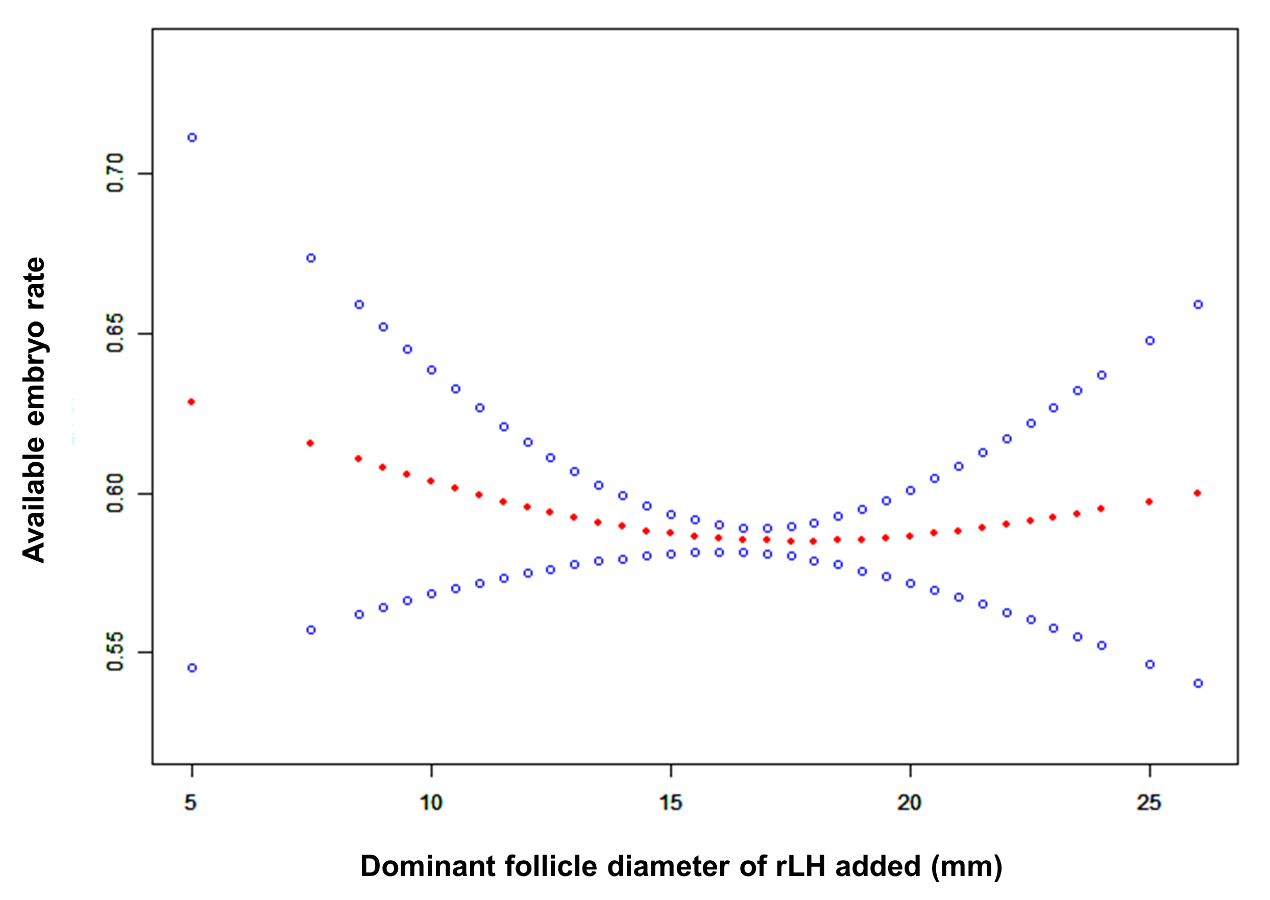


**Figure S1. A smooth fitting curve analysis between dominant follicle diameter when rLH added and** **available embryo rates.**

The illustrated curved line shows the relation between the dominant follicle diameter when rLH added and available embryo rates. The area between two dotted lines is expressed as the 95% CI.
