# Supplementary material for: Effect of recombinant LH supplementation timing on clinical pregnancy outcome in long-acting GnRHa downregulated cycles
Source: BMC Pregnancy Childbirth. 2022 Aug 9;22:632. doi: 10.1186/s12884-022-04963-x (PMC9364622; doi:10.1186/s12884-022-04963-x)
Supplement: Supplementary file 2 — Additional file 2: Table S1. Comparison of different LH afterGnRHa. [file 12884_2022_4963_MOESM2_ESM.docx]

**Table S1.** Comparison of different LH after GnRHa groups.

| **LH after GnRHa (mIU/mL)** | **<0.5 (n=874)** | **≥0.5 (n=1352)** | **P-value** |
| --- | --- | --- | --- |
| **Female age (y)** | 29.76 ± 3.59 | 29.81 ± 3.80 | 0.772 |
| **BMI (kg/m^2^)** | 23.66 ± 3.33 | 22.78 ± 3.13 | <0.001 |
| **Infertility duration (y)** | 3.26 ± 2.12 | 3.28 ± 2.17 | 0.862 |
| **Infertility type** |  |  | 0.766 |
| **Primary infertility** | 484 (55.38%) | 740 (54.73%) |  |
| **Secondary infertility** | 390 (44.62%) | 612 (45.27%) |  |
| **Infertility factors** |  |  | N/A |
| **Tubal factor** | 565 (64.65%) | 931 (68.86%) |  |
| **Ovulatory obstacle** | 179 (20.48%) | 205 (15.16%) |  |
| **Reproductive tract** | 2 (0.230%) | 0 (0.00%) |  |
| **Endometriosis or adenomyosis** | 17 (1.95%) | 32 (2.37%) |  |
| **Male factors** | 72 (8.24%) | 111 (8.21%) |  |
| **Unexplained infertility** | 39 (4.46%) | 73 (5.40%) |  |
| **Basal FSH (mIU/mL)** | 6.97 ± 1.66 | 7.17 ± 1.81 | 0.006 |
| **Basal LH (mIU/mL)** | 5.83 ± 3.85 | 6.38 ± 4.14 | 0.002 |
| **Basal E_2_ (pg/mL)** | 43.70 ± 65.16 | 44.47 ± 40.42 | 0.733 |
| **AFC (n)** | 20.48 ± 5.04 | 20.57 ± 5.37 | 0.687 |
| **Initiated Gn dose (IU)** | 129.19 ± 34.42 | 129.32 ± 34.99 | 0.933 |
| **FSH after GnRHa (mIU/mL)** | 3.34 ± 1.40 | 3.54 ± 1.46 | 0.001 |
| **LH after GnRHa (mIU/mL)** | 0.36 ± 0.09 | 0.82 ± 0.39 | <0.001 |
| **Total Gn dose (IU)** | 2066.96 ± 676.76 | 1901.03 ± 558.92 | <0.001 |
| **Gn duration (y)** | 12.22 ± 2.56 | 11.93 ± 2.26 | 0.004 |
| **E_2_ on hCG day (pg/mL)** | 2557.43 ± 1160.41 | 3109.85 ± 1378.05 | <0.001 |
| **LH on hCG day (mIU/mL)** | 1.30 ± 0.59 | 1.81 ± 0.82 | <0.001 |
| **P on hCG day (ng/mL)** | 0.60 ± 0.39 | 0.67 ± 0.41 | <0.001 |
| **Em (mm)** | 11.98 ± 2.55 | 12.01 ± 2.53 | 0.75 |
| **Total rLH dose (IU)** | 215.73 ± 109.73 | 177.74 ± 94.86 | <0.001 |
| **No. of of retrieved oocytes (n)** | 11.72 ± 3.63 | 12.37 ± 3.70 | <0.001 |
| **No. of MII oocytes (n)** | 10.22 ± 3.50 | 10.84 ± 3.60 | <0.001 |
| **MII rate** | 0.87 ± 0.14 | 0.88 ± 0.13 | 0.526 |
| **No. of fertilized oocytes (n)** | 9.57 ± 3.45 | 10.26 ± 3.58 | <0.001 |
| **No. of normally fertilized oocytes (2PN) (n)** | 8.70 ± 3.32 | 9.29 ± 3.39 | <0.001 |
| **2PN rate** | 0.91 ± 0.11 | 0.91 ± 0.11 | 0.408 |
| **No. of available embryos (n)** | 5.06 ± 2.48 | 5.23 ± 2.47 | 0.121 |
| **Available embryo rate** | 0.59 ± 0.21 | 0.58 ± 0.21 | 0.182 |
| **No. of transferred embryos (n)** | 1.58 ± 0.49 | 1.55 ± 0.50 | 0.189 |
| **Type of transferred embryo** |  |  | 0.01 |
| **cleavage-stage embryo** | 735 (84.10%) | 1078 (79.73%) |  |
| **blastocyst** | 139 (15.90%) | 274 (20.27%) |  |
| **No. of implanted embryos (n)** | 1.29 ± 0.48 | 1.28 ± 0.48 | 0.624 |
| **OHSS rate** | 56 (6.41%) | 127 (9.39%) | 0.081 |
| **Clinical pregnancy rate** | 617 (70.60%) | 970 (71.75%) | 0.558 |
| **Early miscarriage rate** | 36 (5.84%) | 78 (8.04%) | 0.097 |
| **Live birth rate** | 552 (63.16%) | 854 (63.17%) | 0.997 |
