# Supplementary material for: Effect of recombinant LH supplementation timing on clinical pregnancy outcome in long-acting GnRHa downregulated cycles
Source: BMC Pregnancy Childbirth. 2022 Aug 9;22:632. doi: 10.1186/s12884-022-04963-x (PMC9364622; doi:10.1186/s12884-022-04963-x)
Supplement: Supplementary file 3 — Additional file 3: Table S2. Comparison ofdifferent dominant follicle diameter of rLH added groups. [file 12884_2022_4963_MOESM3_ESM.docx]

**Table S2.** Comparison of different dominant follicle diameter of rLH added groups.

| **Dominant follicle diameter of rLH added (mm)** | **<14 (n=214)** | **≥14 (n=2012)** | **P value** |
| --- | --- | --- | --- |
| **Female age (y)** | 30.15 ± 3.81 | 29.75 ± 3.70 | 0.143 |
| **BMI (kg/m^2^)** | 23.49 ± 3.380 | 23.09 ± 3.22 | 0.087 |
| **Infertility duration (y)** | 3.37 ± 2.22 | 3.26 ± 2.14 | 0.502 |
| **Infertility type** |  |  | N/A |
| **Primary infertility** | 108 (50.47%) | 1116 (55.47%) |  |
| **Secondary infertility** | 106 (49.53%) | 896 (44.53%) |  |
| **Infertility factors** |  |  |  |
| **Tubal factor** | 152 (71.03%) | 1344 (66.80%) |  |
| **Ovulatory obstacle** | 36 (16.82%) | 348 (17.30%) |  |
| **Reproductive tract** | 0 (0.00%) | 2 (0.10%) |  |
| **Endometriosis or adenomyosis** | 3 (1.40%) | 46 (2.29%) |  |
| **Male factors** | 10 (4.67%) | 92 (4.57%) |  |
| **Unexplained infertility** | 13 (6.07%) | 180 (8.95%) |  |
| **Basal FSH (mIU/mL)** | 7.49 ± 1.99 | 7.05 ± 1.72 | <0.001 |
| **Basal LH (mIU/mL)** | 6.38 ± 4.08 | 6.14 ± 4.04 | 0.419 |
| **Basal E_2_ (pg/mL)** | 46.77 ± 44.47 | 43.89 ± 52.27 | 0.437 |
| **AFC (n)** | 20.12 ± 4.91 | 20.58 ± 5.28 | 0.225 |
| **Initiated Gn dose (IU)** | 135.11 ± 39.09 | 128.65 ± 34.22 | 0.01 |
| **FSH after GnRHa (mIU/mL)** | 3.35 ± 1.37 | 3.48 ± 1.45 | 0.209 |
| **LH after GnRHa (mIU/mL)** | 0.55 ± 0.38 | 0.65 ± 0.38 | <0.001 |
| **Total Gn dose (IU)** | 2390.95 ± 826.22 | 1921.00 ± 567.82 | <0.001 |
| **Gn duration (y)** | 12.74 ± 3.00 | 11.97 ± 2.30 | <0.001 |
| **E_2_ on hCG day (pg/mL)** | 2781.46 ± 1265.12 | 2904.81 ± 1330.37 | 0.195 |
| **LH on hCG day (mIU/mL)** | 1.48 ± 0.69 | 1.62 ± 0.79 | 0.008 |
| **P on hCG day (ng/mL)** | 0.58 ± 0.37 | 0.65 ± 0.41 | 0.03 |
| **Em (mm)** | 11.98 ± 2.81 | 12.00 ± 2.51 | 0.938 |
| **Total rLH dose (IU)** | 360.28 ± 126.69 | 174.83 ± 81.52 | <0.001 |
| **No. of of retrieved oocytes (n)** | 11.38 ± 3.54 | 12.19 ± 3.69 | 0.002 |
| **No. of MII oocytes (n)** | 10.01 ± 3.39 | 10.66 ± 3.59 | 0.012 |
| **MII rate** | 0.88 ± 0.13 | 0.88 ± 0.13 | 0.654 |
| **No. of fertilized oocytes (n)** | 9.44 ± 3.32 | 10.05 ± 3.56 | 0.017 |
| **No. of normally fertilized oocytes (2PN) (n)** | 8.52 ± 3.16 | 9.12 ± 3.39 | 0.014 |
| **2PN rate** | 0.90 ± 0.11 | 0.91 ± 0.11 | 0.407 |
| **No. of available embryos (n)** | 4.86 ± 2.35 | 5.20 ± 2.49 | 0.054 |
| **Available embryo rate** | 0.59 ± 0.22 | 0.59 ± 0.21 | 0.86 |
| **No. of transferred embryos (n)** | 1.62 ± 0.49 | 1.56 ± 0.50 | 0.094 |
| **Type of transferred embryo** |  |  | 0.007 |
| **cleavage-stage embryo** | 189 (88.32%) | 1624 (80.72%) |  |
| **blastocyst** | 25 (11.68%) | 388 (19.28%) |  |
| **No. of implanted embryos (n)** | 1.24 ± 0.44 | 1.29 ± 0.48 | 0.174 |
| **OHSS rate** | 17 (7.94%) | 166 (8.25%) | 0.739 |
| **Clinical pregnancy rate** | 152 (71.03%) | 1435 (71.32%) | 0.928 |
| **Early miscarriage rate** | 16 (10.53%) | 98 (6.83%) | 0.093 |
| **Live birth rate** | 131 (61.22%) | 1275 (63.37%) | 0.534 |
